# Hospital Accreditation Type and Patient Safety Outcomes: A National Comparison

**DOI:** 10.63116/NVMQ4217

**Published:** 2026-02-13

**Authors:** Magdalynn Kayfes

**Affiliations:** Health Informatics & Information Management, College of St. Scholastica

**Keywords:** hospital accreditation, patient safety outcomes, CMS Care Compare, health informatics, quality measurement, The Joint Commission, DNV Healthcare

## Abstract

**Background:**

Hospital accreditation is widely viewed as a mechanism to promote patient safety and quality improvement. However, the extent to which different accrediting bodies are associated with variation in performance outcomes remains unclear. The authors in this study assessed whether the performance on publicly reported patient safety outcomes in US acute care hospitals with 250 or more licensed beds, accredited by The Joint Commission (TJC) or Det Norske Veritas Healthcare, significantly differs.

**Methods:**

The authors used a retrospective, cross-sectional study design. They merged hospital accreditation and bed count data with publicly available Centers for Medicare & Medicaid Services performance data and included, in total, 1043 hospitals. The authors analyzed 24 outcome measures, including timely and effective care, complications, infections, mortality, readmissions, and the Overall Star Rating. The authors used Welch’s independent 2-sample *t* tests to compare patient safety outcomes between TJC- and Det Norske Veritas–accredited hospitals and the 1-sample *t* tests to assess each group’s performance relative to national benchmarks.

**Results:**

The accreditation groups for 23 of the 24 measures analyzed did not show statistically significant differences. Heart failure mortality was significantly lower in TJC-accredited hospitals (*P* < .05). Both groups outperformed national benchmarks on several infection-related measures, including catheter-associated urinary tract infections, central line–associated bloodstream infections, and *Clostridioides difficile* rates.

**Conclusions:**

Findings show that hospital accreditation type is not strongly associated with differences in patient safety outcomes. Although accreditation remains important for compliance and accountability, broader organizational, operational, and cultural factors may play a greater role in driving safety performance.

## BACKGROUND

Every day, patients enter hospitals expecting to receive safe, high-quality care. Despite decades of policy reform and advances in medical technology, preventable harm continues to affect health care delivery. In the United States, it is estimated that medical errors cause >250,000 deaths annually, making it the third leading cause of death nationally.^1^ Globally, the World Health Organization reports that unsafe care contributes to more than 3 million deaths each year.^2^ As health care increasingly relies on digital reporting tools, informatics platforms such as the Centers for Medicare and Medicaid Services (CMS) Care Compare website play a crucial role in making patient safety data transparent, standardized, and actionable for hospitals and the public. These statistics underscore the urgent need to evaluate the effectiveness of systems designed to safeguard hospital quality, among which one of the most prominent is hospital accreditation.

Hospital accreditation offers external validation that a health care organization meets defined safety and quality standards. The Joint Commission (TJC) is the most widely used accrediting body in the United States, currently accrediting approximately 70% of hospitals, or about 3800 facilities.^3^ Det Norske Veritas (DNV) Healthcare is a newer alternative that accredits more than 350 US hospitals and emphasizes International Organization for Standardization (ISO) 9001 certification, systems thinking, and continuous quality improvement.^4^ Although both accreditors promote patient safety, their models differ: TJC applies a standards-based framework with triennial surveys, whereas DNV conducts annual site visits and requires ISO 9001 compliance.^4,5^

Although accreditation is often perceived as reflecting higher-quality care, evidence on its association with patient outcomes remains mixed. Previous research found no significant differences in 30-day mortality rates between TJC- and state-accredited hospitals and only modest differences in readmission rates.^6^ Another study similarly concluded that accreditation type was not a strong predictor of hospital quality, with characteristics such as teaching status, ownership, and size having more explanatory power.^7^ However, many of these studies rely on nonstandard outcomes and dated datasets or lack specificity in the accreditation mechanisms being evaluated.

In addition to accreditor type, hospital-level structural and cultural factors substantially influence safety performance. Larger hospitals with higher patient volumes often achieve better outcomes because of specialization and procedural expertise.^8^ Financially secure institutions are more capable of investing in staff development, infrastructure, and compliance processes, all of which support performance on quality metrics.^9,10^ In addition, hospitals that foster a strong safety culture and apply high-reliability principles tend to experience lower rates of adverse events.^11,12^ These findings suggest that accreditation may be an important factor, but not sufficient on its own, to drive optimal patient safety outcomes.

Patient safety outcomes represent standardized, risk-adjusted measures of mortality, complications, infections, and readmissions that reflect the actual results of care rather than adherence to process alone.^13^ These metrics are publicly reported to support transparency and allow patients and stakeholders to compare hospital performance. Although these measures do not capture all dimensions of care, they offer a consistent foundation for assessing safety performance across institutions.

Despite the dominance of TJC and the growing presence of DNV, few studies have directly compared CMS-reported patient safety outcomes across accreditor types, particularly among large hospitals where public reporting is most comprehensive. This study addresses that gap by evaluating whether performance across 24 CMS-defined patient safety outcomes based on accreditor differ in US acute care hospitals with 250 or more licensed beds. These include measures of timely and effective care, mortality, complications, infections, readmissions, and the CMS Overall Star Rating.

The authors in this study used CMS Care Compare data, a public-facing informatics tool, to examine how hospital accreditation type relates to measurable patient safety outcomes. As a publicly accessible platform that aggregates standardized quality metrics, CMS Care Compare relies on robust health information processes—from accurate data submission to measure calculation and reporting. Because these data underpin benchmarking, transparency efforts, and organizational decision-making, understanding whether accreditation status aligns with differences in publicly reported outcomes is increasingly relevant for both quality leaders and informatics professionals. As accreditation continues to influence national reporting frameworks and value-based reimbursement policy, assessing whether these designations are associated with meaningful differences in performance is essential. By analyzing a large, standardized dataset, the authors of this study clarified the role of accreditation in federal quality reporting and offers insights that may inform hospital strategy, regulatory evaluation, and future informatics research.

## RESEARCH QUESTIONS AND HYPOTHESES

### Primary Research Question

Do CMS-reported patient safety outcomes differ between US acute care hospitals with 250 or more licensed beds accredited by TJC and those accredited by DNV Healthcare?

### Secondary Aim

To evaluate whether the average performance of hospitals in each accreditor group significantly differs from national CMS reference values for select patient safety outcomes.

### Null Hypothesis

There are no statistically significant differences in CMS-reported patient safety outcomes between hospitals with 250 or more licensed beds accredited by TJC and those accredited by DNV.

#### Alternative hypothesis

There are statistically significant differences in CMS-reported patient safety outcomes between hospitals with 250 or more licensed beds accredited by TJC and those accredited by DNV.

## METHODS

To assess whether the hospital accreditation type is associated with differences in patient safety outcomes, a retrospective, cross-sectional analysis of publicly reported CMS quality data was conducted. The following sections describe the study design, hospital selection criteria, outcome measures, and statistical methods.

### Study Design

This study used a retrospective, cross-sectional, comparative design to assess differences in CMS-reported patient safety outcomes between hospitals accredited by TJC and those by DNV Healthcare. A quantitative approach was used to examine hospital-level performance data across multiple patient safety domains. This design was appropriate given the observational nature of the data and the aim to evaluate group-level differences without introducing experimental intervention. In addition, the CMS Care Compare platform, a publicly accessible digital informatics tool, served as the primary data source, supporting standardized hospital quality comparisons across the United States.

### Hospital Selection Criteria

The study population included US acute care hospitals listed on the CMS Care Compare platform. Hospitals were eligible for inclusion if they were accredited by either TJC or DNV Healthcare, had 250 or more licensed beds, and reported at least 1 applicable CMS-reported patient safety outcome. The 250-bed threshold was chosen to ensure inclusion of hospitals with sufficient patient volume for CMS to publicly report outcome data. Smaller hospitals, including rural and critical access facilities, are often excluded because of small denominators and suppressed metrics.

Accreditation status was verified using each accreditor’s public database. TJC-accredited hospitals were filtered to include only those listed under the “Hospital” accreditation program. For DNV, only facilities certified under the ISO 9001 quality management standard were included because this reflects full accreditation under DNV’s quality framework.

Licensed bed counts were confirmed using the most recent available data from state department of health or hospital association websites. If state-level data were unavailable, facility websites were used to verify bed counts. Hospitals were excluded if they were accredited by an organization other than TJC or DNV, had missing or unverifiable licensed bed count data, or lacked publicly reported CMS data for any of the selected patient safety outcomes.

In total, 1043 hospitals met all inclusion criteria and were included in the final analysis. For DNV Healthcare, 367 hospitals were accredited nationwide; 103 had 250 or more licensed beds, resulting in the exclusion of 264 hospitals based on bed count. Of the 103 eligible hospitals, 97 had publicly reported CMS-reported patient safety outcomes, resulting in an additional exclusion of 6 hospitals. For TJC, 3781 hospitals were accredited nationwide; 1058 met the 250-bed threshold, resulting in the exclusion of 2723 hospitals. Of those 1058 hospitals, 946 had available CMS-reported patient safety outcomes, resulting in the exclusion of an additional 112 hospitals.

Because the analysis included all eligible hospitals rather than a sampled subset, no a priori sample size calculation was required. The large sample size provides adequate power to detect statistically meaningful differences between groups in this observational analysis. Post hoc estimates indicate that the final sample size provided >80% power to detect small-to-moderate differences in mean patient safety outcomes between TJC- and DNV-accredited hospitals.

### Outcome Measures

This study included 24 CMS-defined hospital-level patient safety outcome measures, spanning 5 domains: timely and effective care, complications, infections, mortality, and readmissions. In addition, the CMS Overall Star Rating was included as a composite quality measure.

Timely and effective care included 1 measure: appropriate sepsis care rates. Complication measures included serious complications and those specific to hip and knee replacement procedures. Infection measures included central line–associated bloodstream infections (CLABSI), catheter-associated urinary tract infections (CAUTI), methicillin-resistant *Staphylococcus aureus* (MRSA) infections, *Clostridioides difficile* (*C. difficile*) infections, and surgical-site infections related to colon surgery and abdominal hysterectomies. Mortality outcomes included death rates for patients admitted with conditions such as chronic obstructive pulmonary disease, heart attack, heart failure, pneumonia, and following coronary artery bypass graft surgery. Readmission measures included the hospital-wide readmission rate as well as readmissions specific to chronic obstructive pulmonary disease, heart attack, heart failure, pneumonia, coronary artery bypass graft, and hip/knee replacement. The CMS Overall Star Rating was also included as a composite measure reflecting a hospital’s performance across multiple quality domains.

All outcome measures were evaluated at the hospital level, with each facility contributing a single aggregate value per reported measure. No primary data collection or clinical intervention was performed. All measures were based on the most recent publicly available CMS data as of March 2025.

CMS defines standardized measurement periods for each outcome. Most mortality and readmission measures reflected a 3-year period from July 1, 2020, through June 30, 2023. Complication measures, including surgical-site infections, typically reflected a 2-year reporting period from July 1, 2021, through June 30, 2023. The rate of complications for patients with hip/knee replacement was reported from July 1, 2020, through March 31, 2023. Surveys, questionnaires, or other primary data collection methods were not used in this study.

### Data Management and Analysis

Data cleaning and statistical analyses were conducted using RStudio (version 2024.12.1 + 563). Relevant R packages included tidyverse, janitor, rstatix, broom, and ggpubr. CMS outcome data were imported from a cleaned dataset compiled from the CMS Care Compare website. All columns were reviewed for consistency and appropriate data types.

For hospitals with missing outcome data, observations were excluded from the analysis on a per-variable basis to ensure that results reflected only complete, reported values. TJC- and DNV-accredited hospitals were compared using the Welch independent 2-sample *t* tests, which do not assume equal variances and are appropriate for unequal sample sizes. These tests were conducted separately for each outcome variable to identify statistically significant differences in mean performance between accreditor groups.

In addition to comparing TJC- and DNV-accredited hospital performance, 1-sample *t* tests were conducted to compare each group’s performance to the national benchmark or result reported by CMS for each measure. CMS reference values were defined as national-level mean or benchmark scores published on the Care Compare platform for each corresponding outcome. All statistical tests were 2-tailed, and a significance level of *P* < .05 was used to determine statistical significance.

### Ethical Considerations

This study did not involve human participants or the collection of individually identifiable data. All analyzed data were publicly available through the CMS Care Compare platform. Because the dataset is de-identified and publicly accessible, Institutional Review Board approval was not required.

## RESULTS

In total, 1043 hospitals met all inclusion criteria. Of the 367 DNV-accredited hospitals, 103 had 250 or more licensed beds. After excluding 4 hospitals without publicly available CMS-reported outcomes, 97 DNV-accredited hospitals remained. Among 3781 TJC-accredited hospitals, 1058 met the number of beds threshold and 946 had available CMS-reported patient safety data. Thus, the final analytic sample included 97 DNV-accredited and 946 TJC-accredited hospitals (Table 1).

**Table 1.** Hospital Sample Characteristics

**Accrediting body**	**Total US accredited hospitals**	**Hospitals with** ≥**250 beds**	**Hospitals with CMS safety outcomes**	**Final population included**
DNV Healthcare	367	103	97	97
TJC	3781	1058	946	946

Abbreviations: CMS, Centers for Medicare & Medicaid Services; DNV, Det Norske Veritas; TJC, The Joint Commission.

Hospitals were included if they had 250 or more licensed beds and at least 1 CMS-reported patient safety outcome. Hospitals without reported outcomes were excluded.

### Comparison of Patient Safety Outcomes Between Accreditors

**Figure 1. Comparison of Heart Failure Mortality Between DNV- and TJC-Accredited Hospitals:**
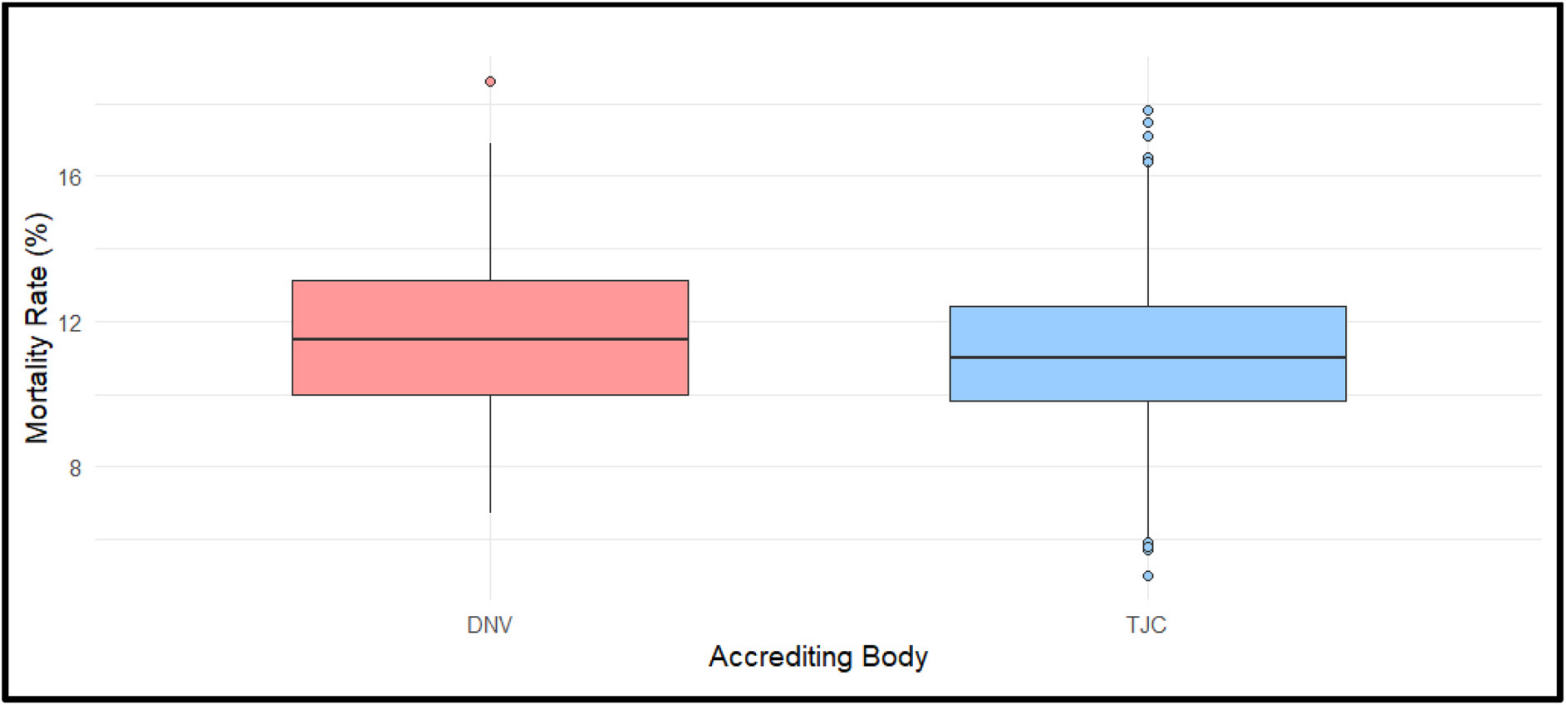
Boxplots show the distribution of heart failure mortality rates for DNV- and TJC-accredited hospitals. Among 24 outcomes analyzed, heart failure mortality was the only outcome that showed a statistically significant difference between groups (*P* = .0167). DNV, Det Norske Veritas; TJC, The Joint Commission.

The Welch independent 2-sample *t* tests were conducted to compare patient safety outcomes between DNV- and TJC-accredited hospitals. Of the 24 outcome measures analyzed (Tables 2 and 3), only heart failure mortality demonstrated a statistically significant difference between groups (*P* = .0167), favoring TJC-accredited hospitals (Figure 1). No other outcome, including sepsis care, complications, infections, or readmissions, demonstrated statistically significant differences between accreditor groups.

**Table 2.** Comparison of Mean Patient Safety Outcomes Between DNV- and TJC-Accredited Hospitals: Sepsis Care, Complications, and Infections

**Domain**	**Patient safety outcome**	**DNV mean**	**TJC mean**	***P* value**	**Statistically significant?**
Timely and effective care	Sepsis care	59.0%	63.0%	.630	No
Complications	Serious complications	1.04	1.01	.227	No
Complications	Rate of complications for hip/knee replacement	3.53%	3.47%	.470	No
Complications	Deaths among patients with serious treatable complications after surgery	181.0	178.0	.279	No
Infections	CLABSI	0.619	0.688	.144	No
Infections	CAUTI	0.579	0.538	.287	No
Infections	SSI—colon surgery	0.847	0.876	.649	No
Infections	SSI—abdominal hysterectomy	1.09	1.10	.945	No
Infections	MRSA bloodstream infection	0.677	0.740	.162	No
Infections	*Clostridioides difficile* infection	0.372	0.377	.834	No

Abbreviations: DNV, Det Norske Veritas; TJC, The Joint Commission; CLABSI, central line–associated bloodstream infections; CAUTI, catheter-associated urinary tract infections; MRSA, methicillin-resistant *Staphylococcus aureus*.

Performance comparisons reflect mean hospital performance for sepsis care, complication rates, and health care-associated infection rates. National results were used for sepsis care and complication measures. National benchmarks were used for infection measures. Statistical significance was assessed using Welch’s *t* test with a threshold of *P* < .05.

**Table 3.** Comparison of Mean Patient Safety Outcomes Between DNV- and TJC-Accredited Hospitals: Mortality, Readmission, and Overall Star Rating

**Domain**	**Patient safety outcome**	**DNV mean**	**TJC mean**	***P* value**	**Statistically significant?**
Mortality	Death rate for COPD patients	9.38%	9.23%	.346	No
Mortality	Death rate for heart attack patients	12.7%	12.5%	.185	No
Mortality	Death rate for heart failure patients	11.7%	11.1%	.0167	Yes
Mortality	Death rate for pneumonia patients	17.7%	17.2%	.121	No
Mortality	Death rate for stroke patients	13.7%	13.5%	.394	No
Mortality	Death rate for CABG surgery patients	3.0%	2.87%	.185	No
Readmissions	Hospital-wide readmission rate	14.8%	14.7%	.267	No
Readmissions	Readmission rate for COPD patients	18.6%	18.6%	.588	No
Readmissions	Readmission rate for heart attack patients	13.7%	13.8%	.630	No
Readmissions	Readmission rate for heart failure patients	20.0%	19.7%	.183	No
Readmissions	Readmission rate for pneumonia patients	16.6%	16.5%	.753	No
Readmissions	Readmission rate for CABG surgery patients	10.9%	10.7%	.0925	No
Readmissions	Readmission rate for hip/knee replacement patients	4.50%	4.58%	.204	No
Overall Star Rating	CMS Overall Star Rating	3.0	3.0	.986	No

Abbreviations: DNV, Det Norske Veritas; TJC, The Joint Commission; CMS, Centers for Medicare & Medicaid Services; CABG, coronary artery bypass graft; COPD, chronic obstructive pulmonary disease.

Performance comparisons reflect mean hospital performance for mortality, readmissions, and CMS Overall Star Rating measures. National results were used as the reference values. Statistical significance was assessed using the Welch *t* test with a threshold of *P* < .05.

### Comparison with National Benchmarks

**Figure 2. Performance Compared With National Reference Values by Outcome and Accrediting Body:**
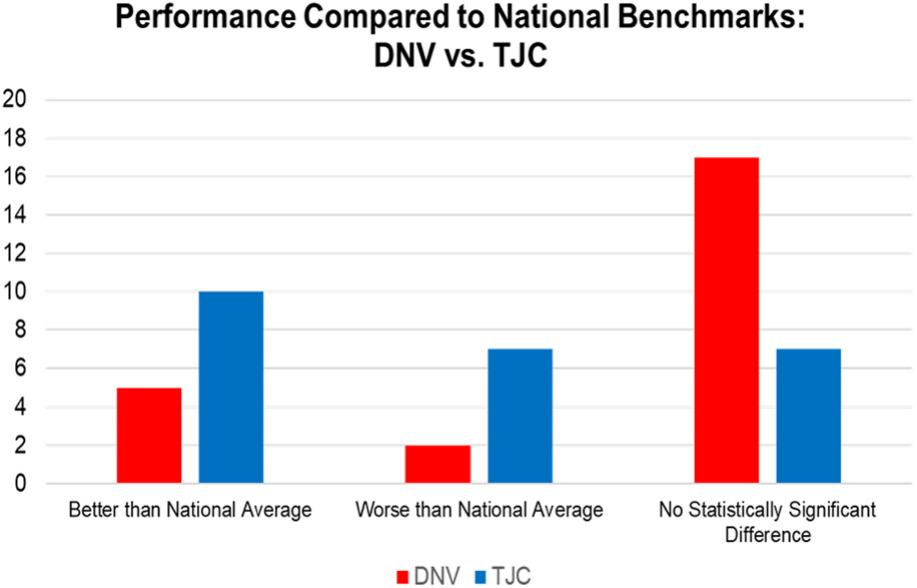
Counts represent the number of patient safety outcomes for which hospital group means were statistically significantly better than, worse than, or not different from national benchmarks or results from the Centers for Medicare & Medicaid Services (*P* < .05).

One-sample *t* tests assessed whether each accreditor group’s mean performance differed from CMS national reference values. As summarized in Table 4, both DNV- and TJC-accredited hospitals significantly outperformed national benchmarks on several infection-related outcomes, including CAUTI and CLABSI and *C. difficile* infections and MRSA bloodstream infections. TJC-accredited hospitals also outperformed national values on multiple mortality and readmission measures. However, compared with DNV-accredited hospitals, TJC-accredited hospitals had a greater number of outcomes that were significantly worse than national values (Figure 2). DNV-accredited hospitals more frequently showed no significant difference from national reference values. Complete statistical results are presented in Appendix A.

**Table 4.** Performance Compared With National Reference Values by Outcome and Accrediting Body

**Domain**	**Outcome**	**National value**	**DNV mean**	**TJC mean**	**DNV comparison to national (α = 0.05)**	**TJC comparison to national (α = 0.05)**
Timely and effective care	Sepsis care	62.0%	59.0%	59.8%	No different	Better
Complications	Serious complications	1.00	1.04	1.01	Worse	Worse
Complications	Rate of complications for hip/knee replacement	3.5%	3.53%	3.47%	No different	No different
Complications	Deaths among patients with serious treatable complications after surgery	176.55	181.0	178.0	No different	No different
Infections	CLABSI	1.00	0.619	0.688	Better	Better
Infections	CAUTI	1.00	0.579	0.538	Better	Better
Infections	SSI—colon surgery	1.00	0.847	0.876	Better	Better
Infections	SSI—abdominal hysterectomy	1.00	1.09	1.10	No different	Worse
Infections	MRSA bloodstream infection	1.00	0.677	0.740	Better	Better
Infections	*Clostridioides difficile* infection	1.00	0.372	0.377	Better	Better
Mortality	Death rate for COPD patients	9.4%	9.38%	9.23%	No different	Better
Mortality	Death rate for heart attack patients	12.6%	12.7%	12.5%	No different	No different
Mortality	Death rate for heart failure patients	11.9%	11.7%	11.1%	No different	Better
Mortality	Death rate for pneumonia patients	17.9%	17.7%	17.2%	No different	Better
Mortality	Death rate for stroke patients	13.9%	13.7%	13.5%	No different	Better
Mortality	Death rate for CABG surgery patients	2.8%	3.03%	2.87%	No different	No different
Readmissions	Hospital-wide readmission rate	14.6%	14.8%	14.7%	Worse	Worse
Readmissions	Readmission rate for COPD patients	18.5%	18.6%	18.6%	No different	Worse
Readmissions	Readmission rate for heart attack patients	13.7%	13.7%	13.8%	No different	Worse
Readmissions	Readmission rate for heart failure patients	19.8%	20.0%	19.7%	No different	No different
Readmissions	Readmission rate for pneumonia patients	16.4%	16.6%	16.5%	No different	Worse
Readmissions	Readmission rate for CABG surgery patients	10.7%	10.9%	10.7%	No different	No different
Readmissions	Readmission rate for hip/knee replacement patients	4.5%	4.5%	4.58%	No different	Worse
Overall Star Rating	CMS Overall Star Rating	NA	3.00	3.00	No different	No different

Abbreviations: DNV, Det Norske Veritas; TJC, The Joint Commission; CMS, Centers for Medicare & Medicaid Services; CLABSI, central line–associated bloodstream infections; CAUTI, catheter-associated urinary tract infections; MRSA, methicillin-resistant *Staphylococcus aureus*; SSI, surgical-site infections; CABG, coronary artery bypass graft; COPD, chronic obstructive pulmonary disease.

CMS national results were used for mortality, complications, and readmission measures, whereas CMS national benchmarks were used for infection-related outcomes. “Better” or “Worse” indicates a statistically significant difference compared with the national reference value (*P* < .05). “No Different” indicates no statistically significant difference.

Overall, these findings indicate limited differences in performance between accreditation types. Both groups demonstrated strengths in infection control. Although TJC-accredited hospitals more frequently outperformed national benchmarks, they also had a greater number of outcomes performing worse than national values. Only heart failure mortality differed significantly between TJC and DNV hospitals.

## DISCUSSION

Contrary to expectations, this study found no consistent differences in CMS-reported patient safety outcomes between large US acute care hospitals accredited by TJC and DNV Healthcare. Of the 24 outcomes analyzed, only heart failure mortality demonstrated a statistically significant difference, favoring TJC-accredited hospitals. These findings suggest that although accreditation reflects compliance with external standards, it may not reliably predict superior performance on federally reported patient safety outcomes.

When evaluating performance against national CMS reference values, neither accreditor demonstrated clear or consistent superiority. Both DNV- and TJC-accredited hospitals performed well on infection-related measures, including CAUTI, CLABSI and MRSA and *C. difficile* infections, yet showed mixed results across mortality and readmission domains. Notably, compared with DNV-accredited hospitals, TJC-accredited hospitals—despite accounting for the majority of US hospitals—had a greater number of outcomes performing worse than national results. This finding challenges long-standing assumptions that accreditation by a more established organization necessarily correlates with higher clinical performance.

These results align with prior research showing limited association between accreditation type and hospital outcomes. Previous studies found no meaningful differences in mortality or readmission between TJC- and state-accredited hospitals, whereas another analysis concluded that organizational characteristics—such as size, teaching status, and ownership—explained more variations in performance than accreditor affiliation.^6,7^ Other studies emphasized the importance of staffing, financial resources, and safety culture, all factors that extend beyond accreditation frameworks.^8,9^

Collectively, these findings reinforce that accreditation, although important for regulatory compliance and public accountability, is only 1 component of hospital quality. Accreditor-specific elements—such as ISO 9001 integration or survey frequency—appear less influential than internal organizational processes, infrastructure, and culture. A 2025 American Hospital Association report similarly found that improvements in teamwork, leadership support, and psychological safety were associated with better patient safety outcomes.^14^

For health information professionals and informatics leaders, these findings have several practical implications. CMS Care Compare is a national informatics platform built on standardized data definitions, automated reporting, and public transparency. The limited differences observed between accreditor groups suggest that the factors influencing outcomes may lie more within internal quality systems of hospitals than within accreditor-specific frameworks. Informatics professionals play a critical role in helping organizations interpret these publicly reported metrics, monitor performance trends, and integrate outcome data into quality dashboards and decision-support tools. By strengthening analytic capacity and aligning internal quality monitoring with federal reporting structures, health information teams can support more targeted, data-driven improvement efforts, regardless of accreditor.

Overall, this study reinforces that accreditation alone does not guarantee superior patient safety outcomes. Future research should continue examining how accreditation interacts with internal quality processes, organizational culture, and informatics infrastructure to influence hospital performance across federally reported quality measures.

## CONCLUSIONS

This study found limited evidence to support the hypothesis that CMS-reported patient safety outcomes differ meaningfully between TJC- and DNV Healthcare–accredited hospitals. Although TJC-accredited hospitals demonstrated a statistically significant advantage in heart failure mortality, the vast majority of outcome measures showed no significant differences between accreditor groups. Both groups outperformed national benchmarks on infection measures, but mixed results across other domains suggest that accreditation status alone is not a reliable predictor of superior patient safety performance.

Several limitations may have influenced these findings. Licensed bed count data were obtained from state- and facility-level sources with varying levels of currency and completeness. The timing of accreditation was not accounted for, meaning some CMS outcomes may reflect care delivered before a hospital’s current accreditor affiliation. In addition, CMS patient safety measures are largely derived from Medicare fee-for-service beneficiaries, which may limit generalizability to broader patient populations. Because TJC accredits the majority of US acute care hospitals, national CMS benchmarks may also disproportionately reflect the performance of TJC-accredited facilities.

Future research could build on this work by examining how internal organizational characteristics—such as revenue, staffing ratios, leadership structures, and safety culture—interact with accreditation frameworks to influence outcomes. Assessing the duration of accreditation and its relationship to performance may also provide additional insight. Expanding analyses to include other accreditor types, including state survey agencies or unaccredited hospitals, could further clarify regulatory impact. Finally, incorporating broader quality domains, such as patient experience, staff engagement, and health equity, may provide a more holistic view of hospital quality.

As health systems continue to rely on federal reporting platforms, such as CMS Care Compare, understanding how accreditation aligns with publicly reported quality outcomes remains important. Identifying the conditions under which accreditation contributes to improved performance—and recognizing where internal processes and informatics infrastructure play a larger role—can help health care leaders design strategies that extend beyond compliance and support meaningful, data-driven improvements in patient safety.

## DISCLOSURES

The authors have nothing to disclose.

## FUNDING

The authors received no funding for this research.

## Appendix

**Table:** Appendix A. Detailed Results of 1-Sample *t* Tests Comparing DNV- and TJC-Accredited Hospitals to National Reference Values

**Patient safety outcome**	**Accrediting body**	***n***	**Group mean**	**National value**	***P* value**	**95% CI lower**	**95% CI upper**
Timely and effective care							
Sepsis care	DNV	96	59.0%	62.0%	.0638	55.9%	62.2%
Sepsis care	TJC	940	59.8%	62.0%	<.001	58.9%	60.8%
Complications							
Serious complications	DNV	97	1.04	1.00	.0493	1.00	1.08
Serious complications	TJC	934	1.01	1.00	.0470	1.00	1.03
Rate of complications for hip/knee replacement	DNV	67	3.53%	3.5%	.694	3.37%	3.7%
Rate of complications for hip/knee replacement	TJC	655	3.47%	3.5%	.245	3.42%	3.52%
Deaths among patients with serious treatable complications after surgery	DNV	89	181.0	176.55	.136	175.0	186.0
Deaths among patients with serious treatable complications after surgery	TJC	839	178.0	176.55	.246	176.0	179.0
Infections							
CLABSI	DNV	94	0.619	1.00	<.001	0.529	0.708
CLABSI	TJC	915	0.688	1.00	<.001	0.659	0.718
CAUTI	DNV	94	0.579	1.00	<.001	0.506	0.652
CAUTI	TJC	925	0.538	1.00	<.001	0.514	0.562
SSI—colon surgery	DNV	91	0.847	1.00	.0105	0.731	0.963
SSI—colon surgery	TJC	864	0.876	1.00	<.001	0.833	0.919
SSI—abdominal hysterectomy	DNV	54	1.09	1.00	.535	0.793	1.40
SSI—abdominal hysterectomy	TJC	444	1.10	1.00	.0223	1.01	1.19
MRSA bloodstream infection	DNV	93	0.677	1.00	<.001	0.596	0.759
MRSA bloodstream infection	TJC	883	0.740	1.00	<.001	0.705	0.776
*C. difficile* infection	DNV	95	0.372	1.00	<.001	0.328	0.416
*C. difficile* infection	TJC	910	0.377	1.00	<.001	0.362	0.392
Mortality							
Death rate for COPD patients	DNV	92	9.38%	9.4%	.918	9.07%	9.7%
Death rate for COPD patients	TJC	885	9.23%	9.4%	.0011	9.12%	9.33%
Death rate for heart attack patients	DNV	90	12.7%	12.6%	.359	12.4%	13.0%
Death rate for heart attack patients	TJC	867	12.5%	12.6%	.189	12.4%	12.6%
Death rate for heart failure patients	DNV	96	11.7%	11.9%	.385	11.2%	12.2%
Death rate for heart failure patients	TJC	922	11.1%	11.9%	<.001	11.0%	11.2%
Death rate for pneumonia patients	DNV	95	17.7%	17.9%	.407	17.1%	18.2%
Death rate for pneumonia patients	TJC	923	17.2%	17.9%	<.001	17.0%	17.4%
Death rate for stroke patients	DNV	92	13.7%	13.9%	.340	13.3%	14.1%
Death rate for stroke patients	TJC	865	13.5%	13.9%`	<.001	13.4%	13.6%
Death rate for CABG surgery patients	DNV	66	3.03%	2.8%	.0534	2.8%	3.27%
Death rate for CABG surgery patients	TJC	592	2.87%	2.8%	.0789	2.79%	2.94%
Readmissions							
Hospital-wide readmission rate	DNV	96	14.8%	14.6%	.0148	14.6%	14.9%
Hospital-wide readmission rate	TJC	939	14.7%	14.6%	.00113	14.6%	14.8%
Readmission rate for COPD patients	DNV	93	18.6%	18.5%	.514	18.4%	18.8%
Readmission rate for COPD patients	TJC	889	18.6%	18.5%	<.001	18.6%	18.7%
Readmission rate for heart attack patients	DNV	87	13.7%	13.7%	.762	13.5%	13.9%
Readmission rate for heart attack patients	TJC	851	13.8%	13.7%	.0159	13.7%	13.9%
Readmission rate for heart failure patients	DNV	96	20.0%	19.8%	.291	19.7%	20.3%
Readmission rate for heart failure patients	TJC	925	19.7%	19.8%	.255	19.7%	19.8%
Readmission rate for pneumonia patients	DMV	95	16.6%	16.4%	.186	16.3%	16.8%
Readmission rate for pneumonia patients	TJC	923	16.5%	16.4%	<.001	16.5%	16.6%
Readmission rate for CABG surgery patients	DNV	66	10.9%	10.7%	.126	10.7%	11.1%
Readmission rate for CABG surgery patients	TJC	591	10.7%	10.7%	.462	10.6%	10.7%
Readmission rate for hip/knee replacement patients	DNV	69	4.5%	4.5%	.963	4.37%	4.62%
Readmission rate for hip/knee replacement patients	TJC	669	4.58%	4.5%	.00317	4.53%	4.64%
Overall Star Rating							
CMS Overall Star Rating	DNV	96	3.00	NA	.986	2.76	3.24
CMS Overall Star Rating	TJC	936	3.00	NA	.986	2.93	3.07

Abbreviations: DNV, Det Norske Veritas; TJC, The Joint Commission; CMS, Centers for Medicare & Medicaid Services; CLABSI, central line–associated bloodstream infections; CAUTI, catheter-associated urinary tract infections; MRSA, methicillin-resistant *Staphylococcus aureus*; SSI, surgical-site infections; CABG, coronary artery bypass graft; COPD, chronic obstructive pulmonary disease; *C. difficile*, *Clostridioides difficile*.

## References

[1] Makary MA, Daniel M. Medical error—the third leading cause of death in the US. BMJ. 2016;353:i2139. doi: 10.1136/bmj.i2139. 27143499

[2] World Health Organization Patient safety September 11, 2023. Accessed August 19, 2025. https://www.who.int/news-room/fact-sheets/detail/patient-safety.

[3] The Joint Commission Hospital accreditation fact sheet. 2025. Accessed August 19, 2025. https://www.jointcommission.org/resources/news-and-multimedia/fact-sheets/facts-about-hospital-accreditation/.

[4] DNV ISO 9001 certification Quality management system. Accessed August 19, 2025. https://www.dnv.com/services/iso-9001-quality-management-3283/.

[5] Wrzesniewski C. Quality and safety through compliance with The Joint Commission Requirements. Calif J Health Syst Pharm. 2017;29(3):61-69. https://research-ebsco-com.akin.css.edu/c/ky7pgv/viewer/pdf/tarpmtcuxf.

[6] Lam MB, Figueroa JF, Feyman Y, Reimold KE, Orav EJ, Jha AK. Association between patient outcomes and accreditation in US hospitals observational study. BMJ. 2018;363:k4011. doi: 10.1136/bmj.k4011. 30337294 PMC6193202

[7] Kato M, Zikos D. Association between hospital accrediting agencies and hospital outcomes of care in the United States. J Hosp Manag Health Policy. 2022;6(12):12-12. doi: 10.21037/jhmhp-21-24.

[8] Mesman R, Westert G, Berden B, Faber M. Why do highvolume hospitals achieve better outcomes? A systematic review about intermediate factors in volumeoutcome relationships. Health Policy. 2015;119(8):1055-1067. https://pure.uvt.nl/ws/portalfiles/portal/18605821/Mesman_Safety_13_10_2017.pdf. doi:10.1016/j.healthpol.2015.04.005. 25958187

[9] Akinleye D, McNutt L, Lazariu V, McLaughlin C. Correlation between hospital finances and quality and safety of patient care. PLoS ONE. 2019;14(8):e0219124. doi: 10.1371/journal.pone.0219124. 31419227 PMC6697357

[10] Encinosa W, Bernard D. Hospital finances and patient safety outcomes. INQUIRY. 2005;42(1):60-72. doi: 10.5034/inquiryjrnl_42.1.60. 16013586

[11] Murray J, Clifford J, Larson S, Lee J, Sculli G. Implementing just culture to improve patient safety. Mil Med. 2023;188(7–8):1596-1599. doi: 10.1093/milmed/usac115. 35587381

[12] Campione J, Famolaro T. Promising practices for improving hospital patient safety culture. Jt Comm J Qual Patient Saf. 2018;44(1):23-32. doi: 10.1016/j.jcjq.2017.09.001. 29290243

[13] Centers for Medicare & Medicaid Services Inpatient measures. U.S. Department of Health & Human Services. June 3, 2025. Accessed August 19, 2025. https://www.cms.gov/medicare/quality/initiatives/hospital-quality-initiative/inpatient-measures.

[14] American Hospital Association Improvement in safety culture linked to better patient and staff outcomes. March 2025 Accessed August 19, 2025. https://www.aha.org/guidesreports/2025-03-11-improvement-safety-culture-linked-better-patient-and-staff-outcomes.

